# Expression of concern: A chaperonin subunit with unique structures is essential for folding of a specific substrate

**DOI:** 10.1371/journal.pbio.3000972

**Published:** 2020-10-20

**Authors:** The PLOS Biology Editors

Following the publication of this article [1], concerns were raised regarding irregularities in Figs 2B, 2C, 3A, 3B, 3C, 4B, 4F, 4G, 5B and S1B. Specifically,

- Concerns were raised about discontinuities in the lower regions of lanes 1 and 5 in Fig 2B, which suggest that rectangular portions of the image were spliced or otherwise altered. The corresponding author provided the underlying gel image for Fig 2B (S1 File). Coomassie Brilliant Blue staining in the areas of concern was not as strong in the raw image as in the figure, supporting that the image was selectively enhanced or otherwise manipulated in these areas. This is in violation of community standards and *PLOS Biology*’s requirements for image data reporting, which require that any adjustments must be applied consistently to the entire image. The underlying data provided by the authors support the overall results presented in Fig 2B, although the *PLOS Biology* Editors remain concerned about the integrity with which the data were reported in the published article.
- Splicing concerns were raised for several figures in this article, including panels of Figs 3A, 3B, 3C and 4B, and panels in the S1B File of the original article. The authors noted that lanes had been removed and/or rearranged during preparation of the panels in questions, as was confirmed by the original raw images provided upon request and presented in the Supporting Information S3–S6 and S9 Files below.
- Vertical discontinuities were detected in several panels in this article, including panels of Figs 2C, 4F, 4G and 5B. The authors noted for that these figures lanes had also been removed and/or rearranged during preparation of the panels in question, but that the original image data were no longer available for these panels. Instead, the authors have provided data from replicate experiments in the Supporting Information S2, S7, S8 and S10 Files below that seemingly support their conclusions.

The underlying data and repeat experiment data received by the journal are published in the supporting information files. The authors have not commented on the availability of underlying data to support the other results reported in the article.

Although overall the data provided to address these concerns seem to support the conclusions of the article, given that original files were not available for assessment in a number of instances and the integrity issues surrounding the preparation of Fig 2B, the *PLOS Biology* Editors issue this Expression of Concern to notify readers and relay the supporting data and updated figures provided by the corresponding author.

## Supporting information

S1 File Original blot underlying the BN-PAGE analysis of thylakoid protein complexes in Fig 2B [1]. (TIF)

S2 File Alternative immunoblot analysis of NDH18 in various genetic backgrounds. (TIF)

S3 File Scan of original blot images underlying the Cpn60β4-HA, Cpn60α, Cpn60β, RbcL, and Cyt f panels presented in Fig 3A [1]. (TIF)

S4 File Scan of the original blot underlying the RbcL panel of Fig 3B [1]. (TIF)

S5 File Scan of the original blot underlying Fig 3C [1]. (TIF)

S6 File Scan of the original blot underlying the Cyt f panel of Fig 4B [1], and repeat experiment blots for protein blot analysis of NdhH and Cpn60β4-HA using Cyt f and RbcL as loading controls. (TIF)

S7 File Scan of repeat experiment results of immunoblot analysis of NdhH and Cpn60β4 from crr27-1 expressing the AtCpn60β4(CM)-HA protein Cyt f and RbcL as loading controls. (TIF)

S8 File Scan of repeat experiment results of protein blot analysis of NdhH and Cpn60β4. (TIF)

S9 File Scan of the original blot underlying the NdhH and Cyt f panels of Fig 5B [1], and repeat experiment blots demonstrating immunodetection of chloroplast proteins from WT, crr27-1, crr27-1 complemented by AtCpn60β4-HA, and three lines of crr27-1 complemented by MpCpn60β-HA (L1–L3). (TIF)

S10 File Scan of the original blots underlying the protein blot analysis of chloroplast proteins presented in S1 Fig of the published article [1]. (TIF)
