# Supplementary figures and images for: Expression of concern: A chaperonin subunit with unique structures is essential for folding of a specific substrate
Source: PLoS Biol. 2020 Oct 20;18(10):e3000972. doi: 10.1371/journal.pbio.3000972 (PMC7575096; doi:10.1371/journal.pbio.3000972)

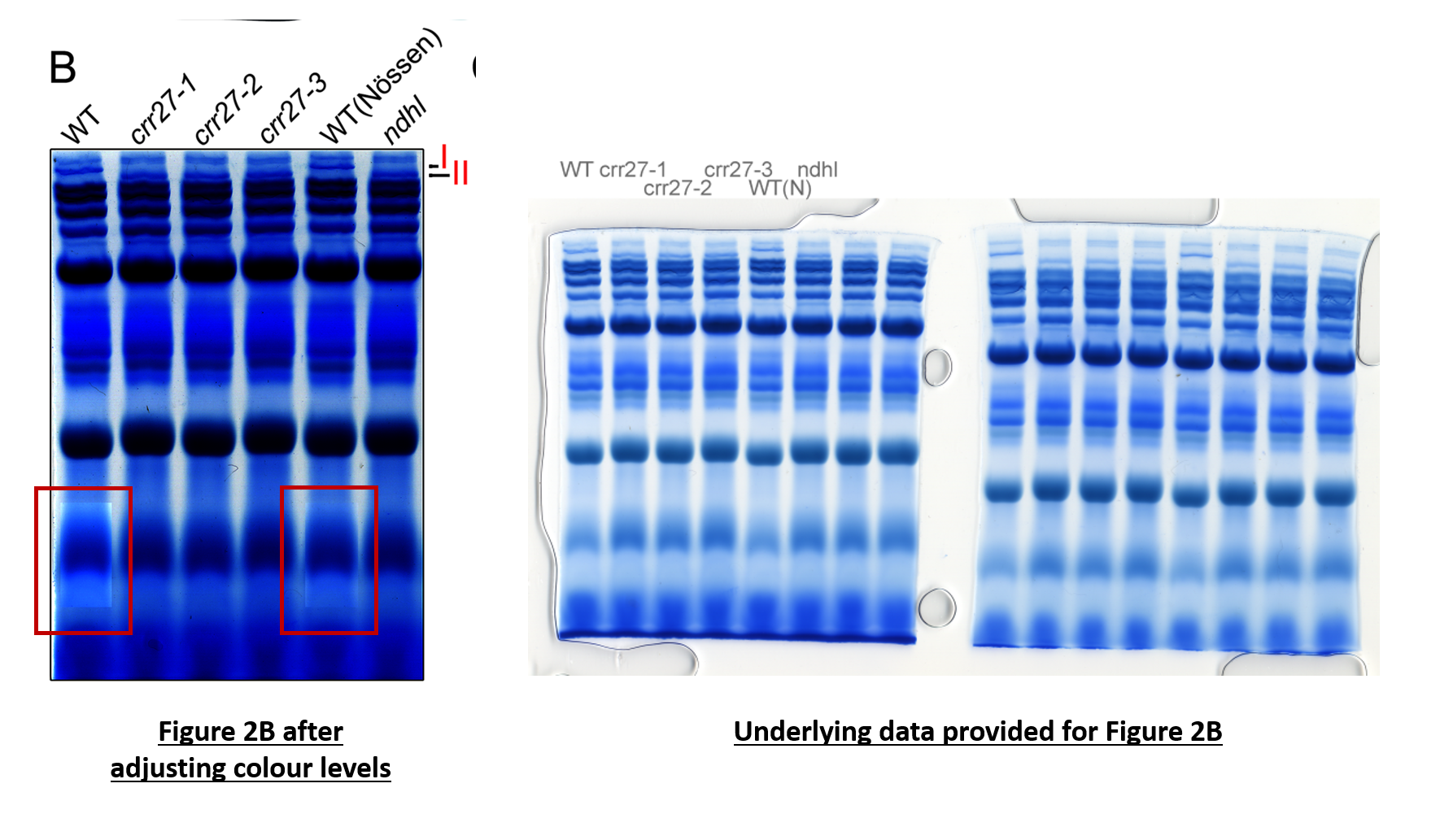

Supplement: S1 File — (TIF) [file pbio.3000972.s001.tif]

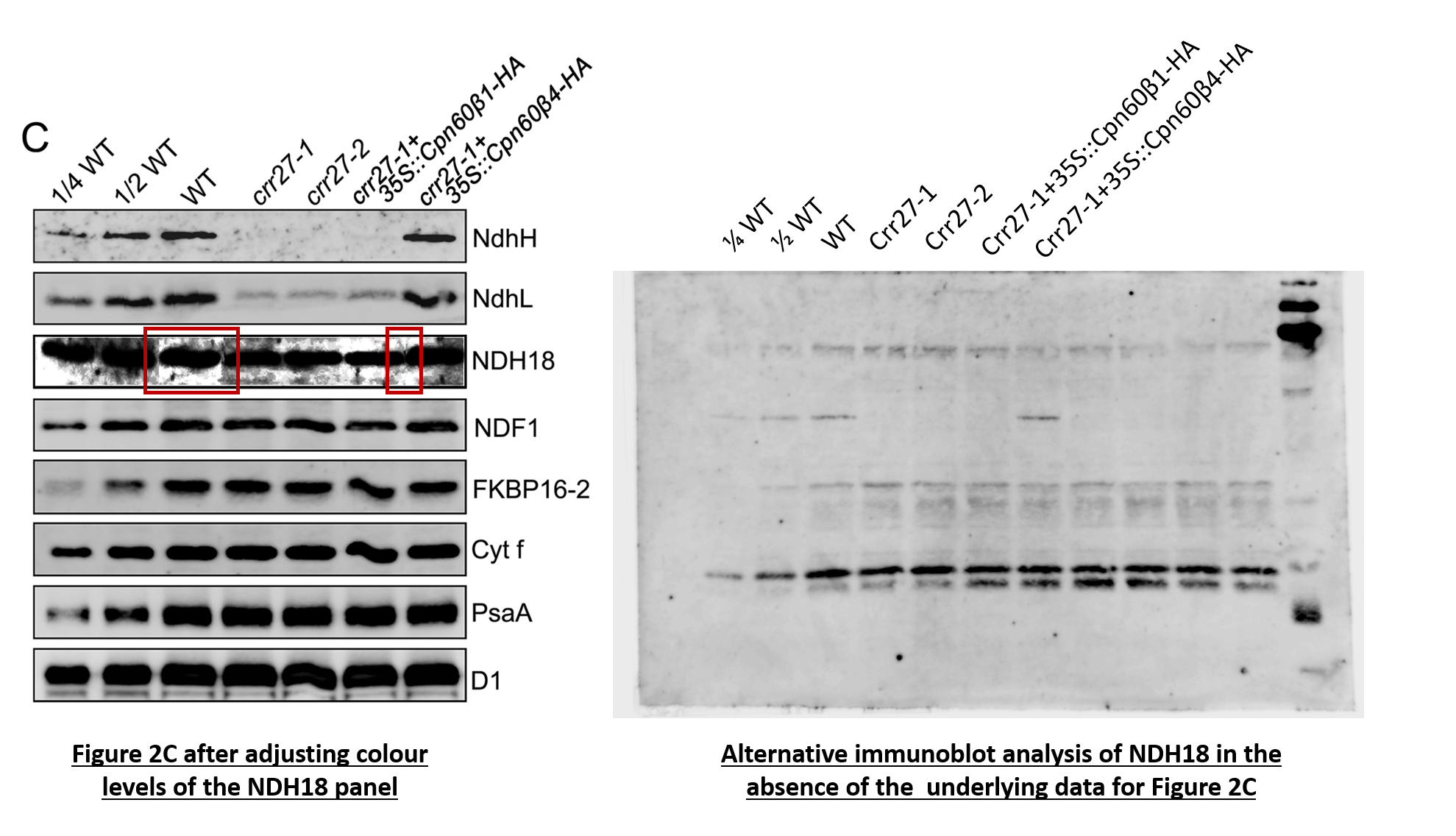

Supplement: S2 File — (TIF) [file pbio.3000972.s002.tif]

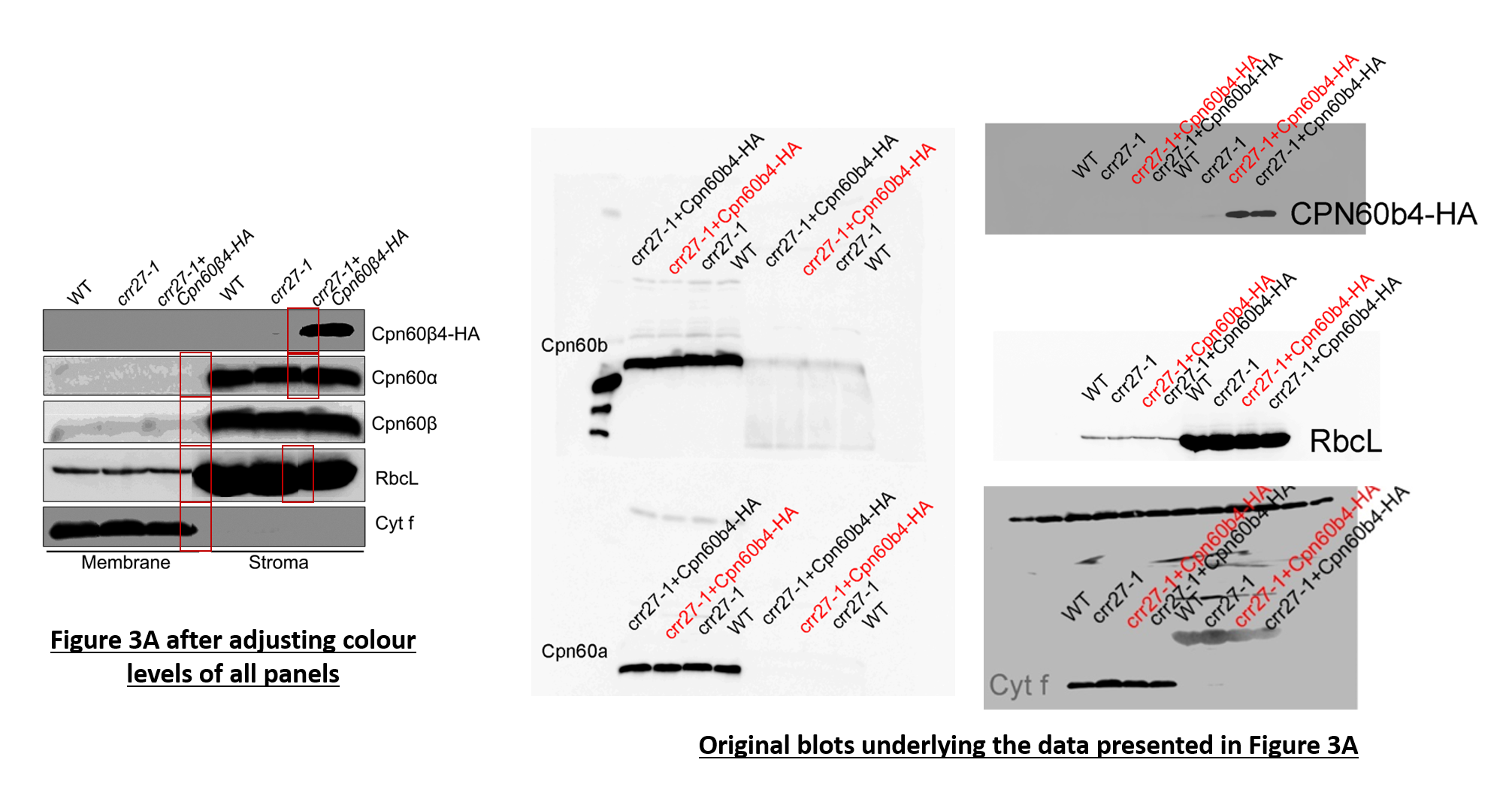

Supplement: S3 File — (TIF) [file pbio.3000972.s003.tif]

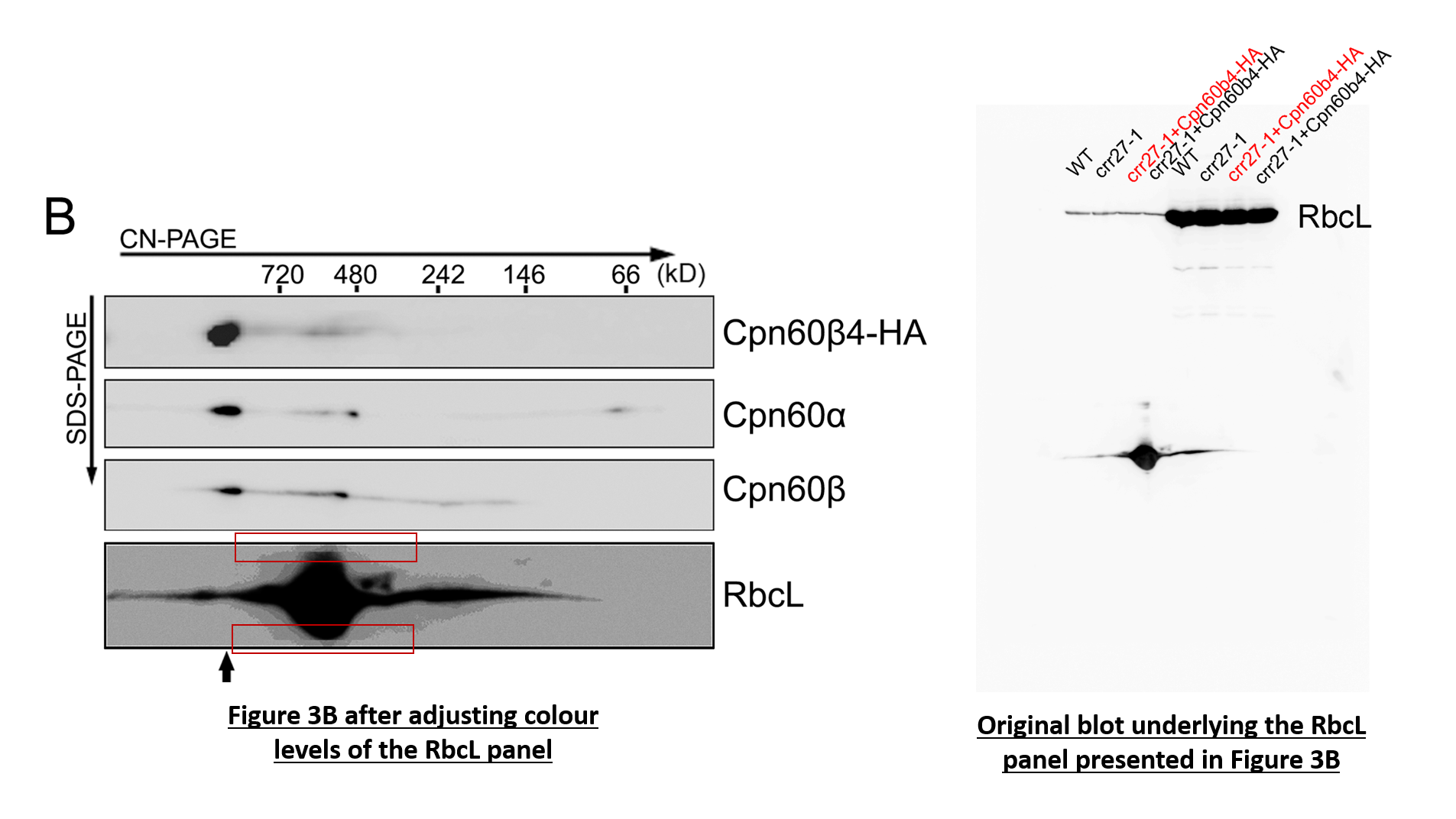

Supplement: S4 File — (TIF) [file pbio.3000972.s004.tif]

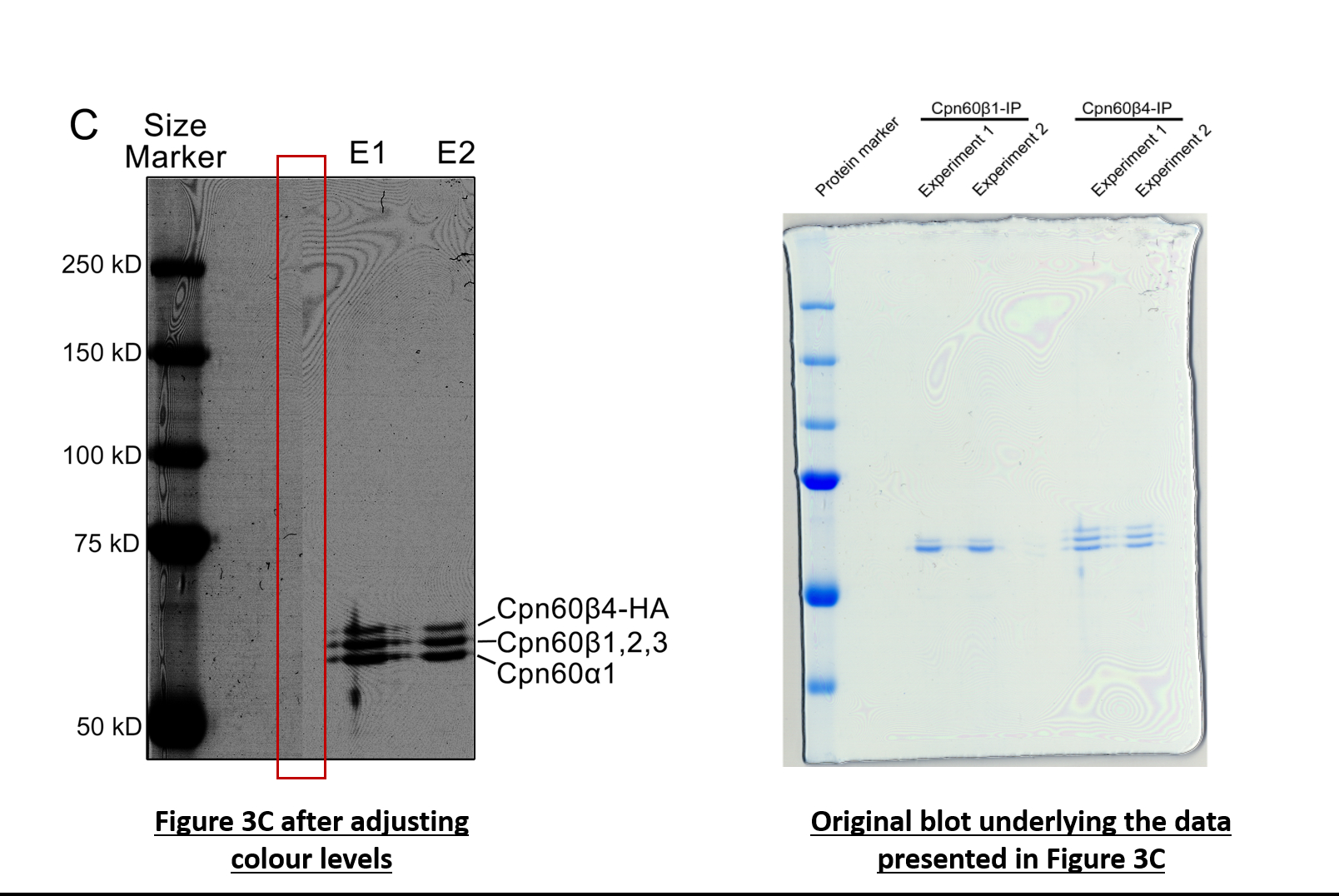

Supplement: S5 File — (TIF) [file pbio.3000972.s005.tif]

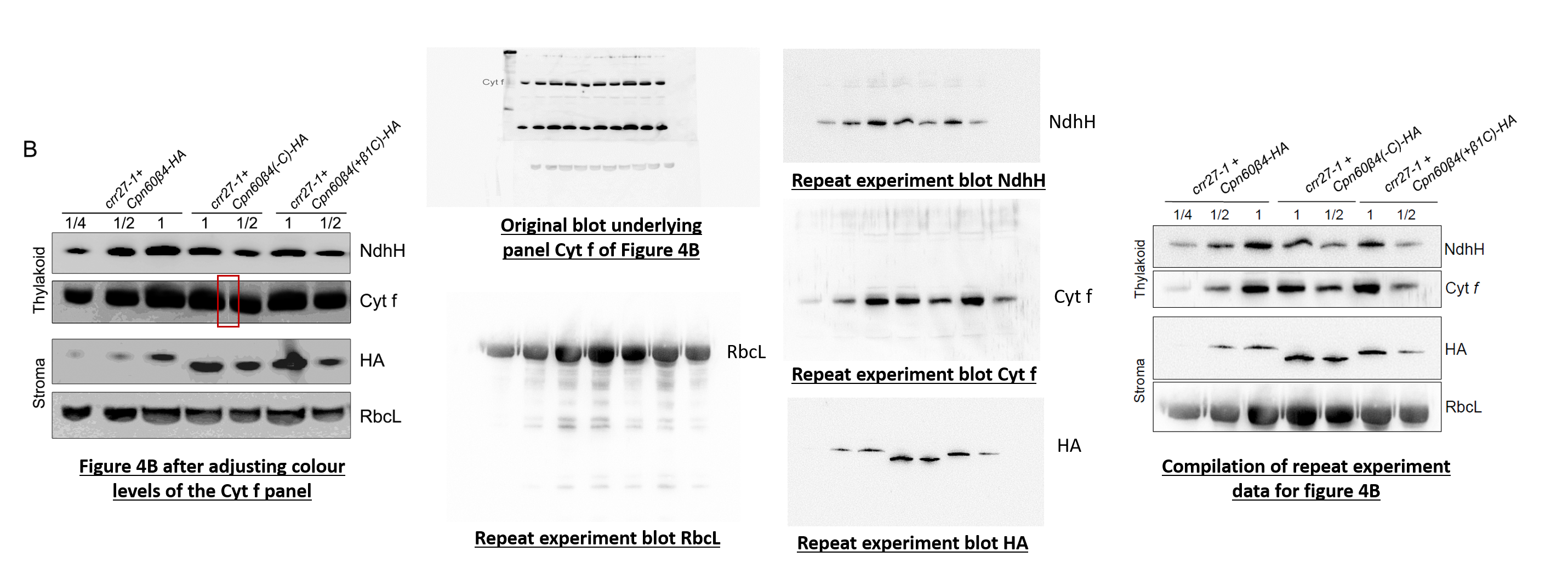

Supplement: S6 File — (TIF) [file pbio.3000972.s006.tif]

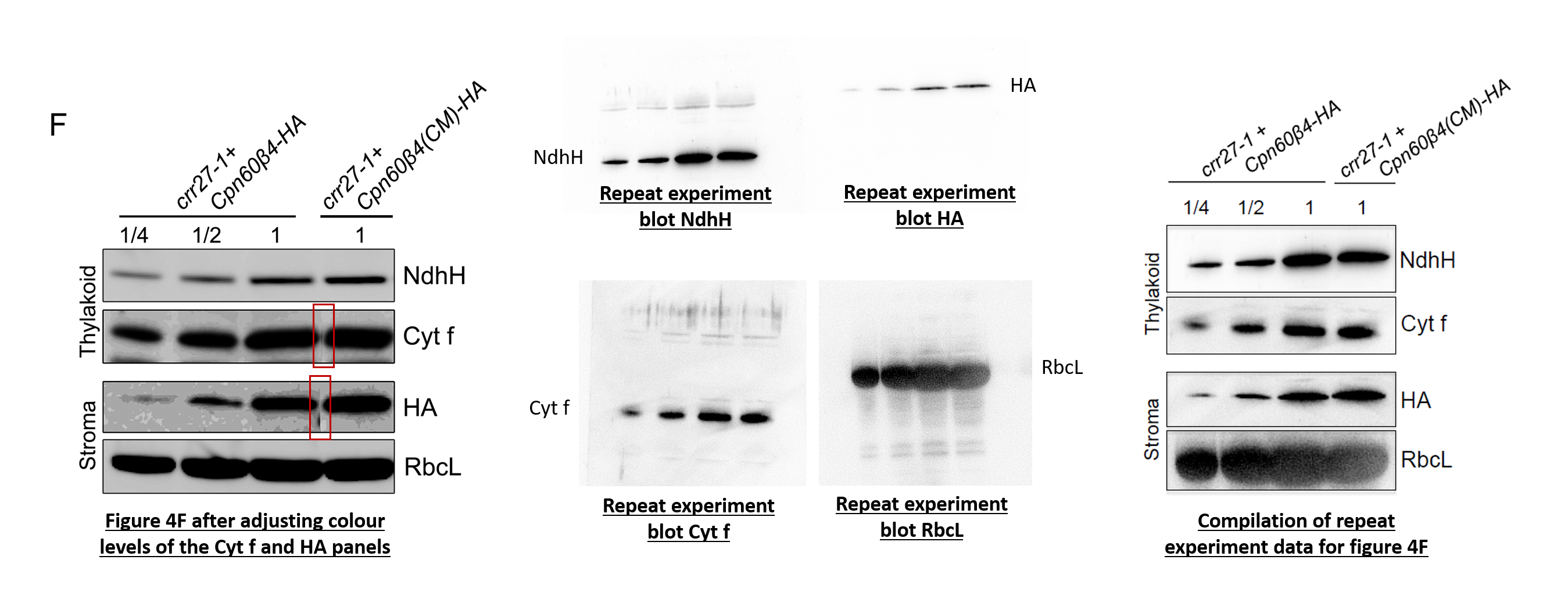

Supplement: S7 File — (TIF) [file pbio.3000972.s007.tif]

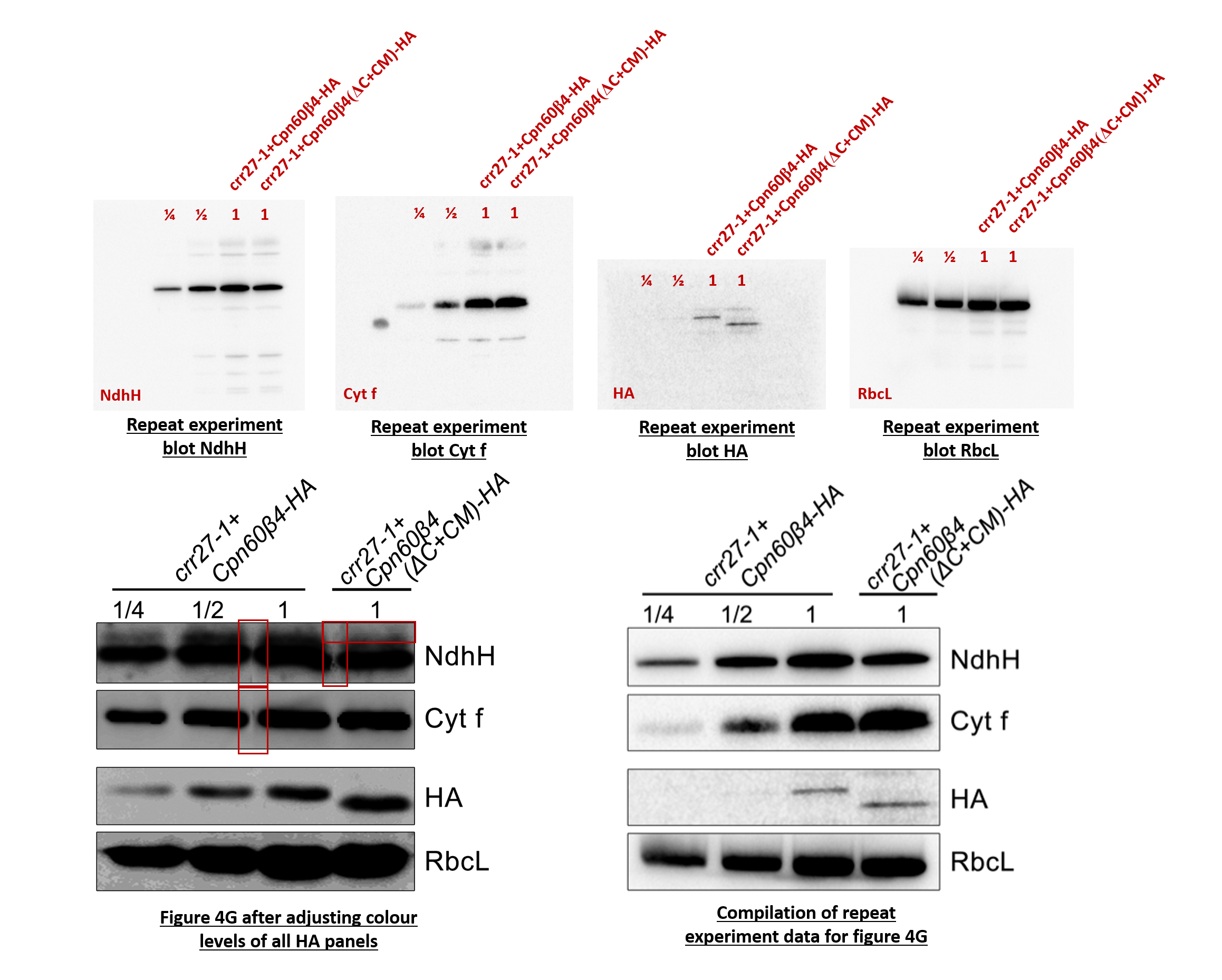

Supplement: S8 File — (TIF) [file pbio.3000972.s008.tif]

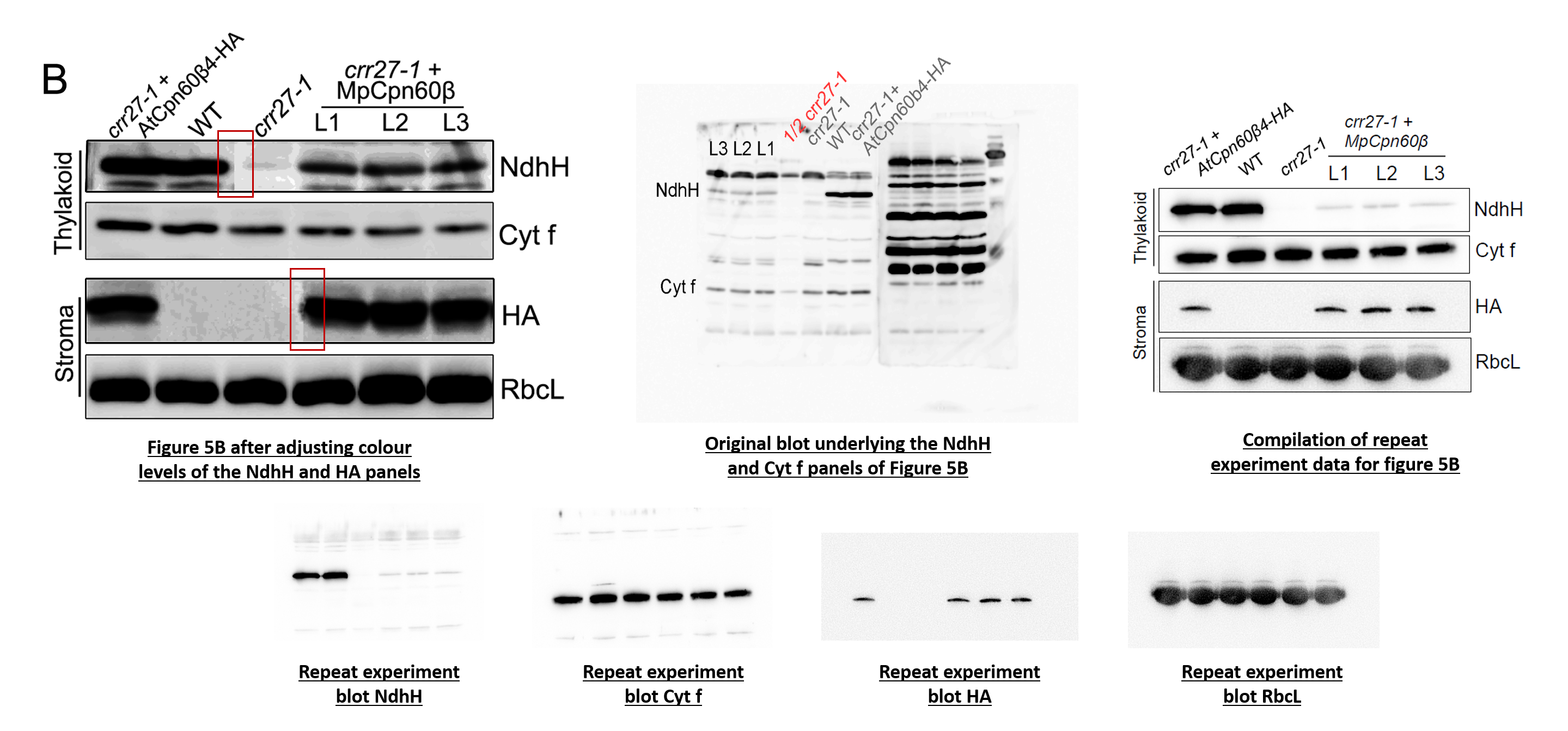

Supplement: S9 File — (TIF) [file pbio.3000972.s009.tif]

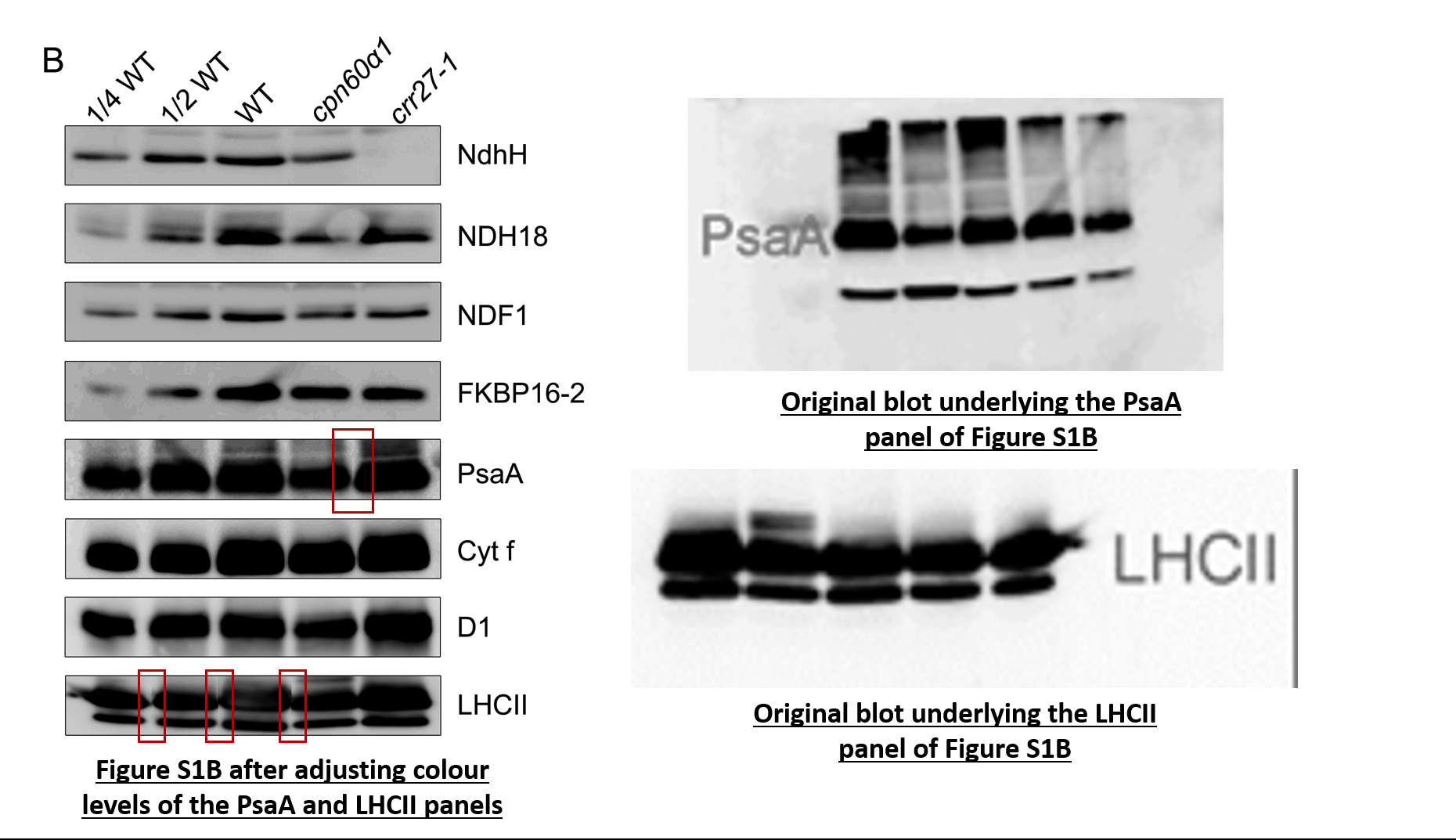

Supplement: S10 File — (TIF) [file pbio.3000972.s010.tif]
